# Tinkering with genes and embryos: the multiple invention of transgenic mice c. 1980

**DOI:** 10.1080/07341512.2019.1694126

**Published:** 2020-01-27

**Authors:** Dmitriy Myelnikov

**Affiliations:** Center for the History of Science, Technology, and Medicine, University of Manchester, Manchester, UK

**Keywords:** Transgenic mice, tinkering, infrastructure, genetic engineering, molecular biology, developmental biology

## Abstract

Genetically modified or ‘transgenic’ mice are a routine experimental tool in biomedical research, commonly produced by injecting DNA into one-cell embryos. These animals were independently invented in 1980 by multiple university groups in the United States and Europe that combined expertise in mouse developmental biology and recombinant DNA techniques, or ‘genetic engineering’. In this article, I examine this multiple invention and argue that research strategies, experimental practices, and funding arrangements that led to transgenic mice are best described as tinkering. These creative and speculative endeavors, combined with partial knowledge of what was happening in competing laboratories, created a fruitful atmosphere for research which led to the multiple invention. The tinkering was, however, underpinned by infrastructures that were crucial to success, some long established, such as mouse supply or embryological tools, and some emerging, such as the informal exchange of isolated genes.

Genetically modified, or transgenic, mice have become a routine tool in biomedical laboratories across the world, and while the overall use of lab animals has steadily declined since the 1980s, the number of transgenic rodents keeps growing.^1^ Mice had been domesticated for genetic research in the early twentieth century, and were heavily standardized and used on a mass scale, but transgenic technology expanded the appeal and reach of these animals.^2^ Made to order in academic core facilities and by commercial breeders, transgenic mice exemplify the technological production of organisms in the service of the life sciences, and embody the promise of biotechnology to improve human health. A transgenic mouse – the ‘Harvard mouse’ modified with an oncogene to induce breast cancer – was the first vertebrate to be awarded a US patent in 1988, and subsequently marketed as OncoMouse™ by the chemical giant DuPont. In the year of the patent, *Fortune* magazine announced the mouse among its products of the year.^3^

Yet despite the prominence of transgenic animals in biotechnological imagination, and their adoption at the key turning point in mergers between the biotech industry and academic biology, transgenic mice were invented in academic settings, through a number of parallel efforts. Between 1980 and 1981, six groups published articles claiming that foreign DNA could be injected into a mouse embryo and persist through development. They relied on a similar method: direct microinjection of DNA solution into a mouse embryo at the one-cell stage, shortly after fertilization. As all participants have agreed, the results were achieved independently, with one exception in which techniques were learned directly from another group. These mice thus paralleled what Robert Merton called a multiple discovery, for him a prime site to investigate scientific norms.^4^ These mice were more inventions than discoveries, but the boundary between the two terms is blurry, a clear case of technoscience.

Multiple inventions are assigned as such retrospectively, and communication to both scientific and lay audiences played a crucial role.^5^ Multiple scientific papers and media accounts appeared within just over a year, and they gave credibility to the new animals and made them an exciting technology to engage with. But how did very similar scientific programmes come to be pursued at multiple locations? What explained the scientific *Zeitgeist*? The similarity between the final publications conceals a diversity of experimental approaches and research agendas that each group pursued. In this article, I explore the practical worlds of the laboratories and collaborations that worked on introducing DNA into mouse embryos, their traditions and diverse experimental expertise, and the ways they arrived at a new kind of animal.

Attempts to move genes between organisms have a long history in various species, but mice and other mammals had remained a distant goal.^6^ As late as 1979 it was not at all clear that introducing DNA into a mouse would work in the near future, or indeed that it was necessarily a productive avenue to pursue. While the ‘cut-and-paste’ tools of recombinant DNA developed in the 1970s played a major role, these experiments were not straightforward applications of a new toolset. Rather, the new mice were made possible by a mutual attraction between developmental biologists eager to exploit the new molecular tools, and molecular biologists excited about working with mammals. This affinity between developmental and molecular biology had multiple precedents in other species and cellular systems, but working with mice promised uncertain but attractive medical applications.^7^ On the level of practice, the cross-disciplinary collaborations also meant aligning two kinds of technical set-ups: the techniques of embryo micromanipulation and the tools for isolating and detecting genes.

This article introduces numerous groups with different commitments and backgrounds within biology – a difference that can be flattened in retrospective accounts, yet significant for the diversity of strategies and motivations. Frank Ruddle’s group at Yale came from somatic cell genetics, a field that aimed to study genes, chromosomes, and heredity in somatic (i.e. not sexual) cells that had been cultured *in vitro*, rather than by observing reproducing organisms. Both Beatrice Mintz at the Fox Chase Institute for Cancer Research and Ralph Brinster at the University of Pennsylvania came from developmental biology, concerned with multidisciplinary study of embryonic development. Mintz had had a stronger interest in genetics and using genes as markers, whereas Brinster had more of a commitment to understanding embryo biochemistry, and to the reproductive sciences. Brinster’s long-distance collaborator, Richard Palmiter at the University of Washington in Seattle, was interested in molecular biology and gene expression, especially in mammalian cells. Thomas Wagner from Ohio University also came from a molecular biology and physical chemistry background, and his experiments were motivated by trying to elucidate the packaging of sperm DNA. His collaborator, Peter Hoppe from the Jackson Laboratory, a stronghold of mouse genetics, was a mouse embryologist. Some groups merged the embryological and molecular aspects by hiring expert postdocs; others established productive long-distance collaborations through well-managed divisions of labor.

I describe these groups’ engagements with embryos and genes as tinkering, a term usually resolved for amateurs engaging with technologies such as radios or rockets, or a jack-of-all-trade. Tinkering was, on occasion, an actor’s category in the late 1970s to minimize the sinister impact of ‘genetic engineering’ and highlight the uncertainty and minimal intervention of recombinant DNA experiments. While these uses were political (and engineering has a fair amount of tinkering involved), they reflected important aspects of laboratory practice. As I discuss below, the eminent molecular biologist and philosopher François Jacob adopted Lévi–Strauss’s *bricolage*, which can be roughly translated as tinkering, to refer to evolution and its modus operandi, and more implicitly to genetic manipulation.

In Leví-Strauss’s *The Savage Mind*, bricolage stood for a ‘science that we prefer to call “prior” rather than “primitive”’, and especially mythical thought ‘that expresses itself by means of heterogeneous repertoire’.^8^ But like Jacob, historians and sociologists of science have extended the notion to the practices of modern science. Karen Knorr Cetina has emphasized tinkering as a key mode of work within scientific laboratories, as opposed to clear and premeditated hypothesis-testing.^9^ More recently, Helen Curry has highlighted the importance of tinkering in attempts to make new plant varieties in the mid-twentieth century across industrial science and amateur horticulture.^10^ The sociologist and photographer Douglas Harper, in his study of knowledge and community at a rural mechanical workshop, offers a reading of bricolage that resonates with my study of embryological and molecular biological skills in laboratories. To tinker is to consider what is available and how it can be reused, while and ‘the set of tools and materials is the sum of all previous projects [that will be] enlarged once again with the materials left over from the current project’.^11^ Similarly, both molecular and embryological tools required ad hoc adjustments, unexpected combinations and honing, and existing resources were essential in developing new experiments. While working towards inserting genes into mice, scientists I discuss sought opportunities to reuse existing samples, tools, and funds. They manufactured their own micropipettes and glass needles, adapted micromanipulation set-ups to accommodate embryos, used available gene samples and secured new ones. A tinkering mode of work could be contrasted with the expansion of Big Biology, and even as transgenic mice were widely adopted in the 1980s and eventually became routine, they remained what one scientist called a ‘boutique operation’, recalcitrant to mass production, high-throughput methods, or efficient standardization.

Histories of biology and technology have long been in productive conversation, but combining the two traditions enables new ways of aligning local accounts of practice with global exchanges, trends, and infrastructures. While the individual scientists tinkered with genes and embryos, they also relied on major infrastructures of academic biology, and lack of access to reliable facilities, instruments and exchange networks could easily translate into failure. The underlying infrastructures, as well as informal communication of what might be possible, played crucial role in creating transgenic mice. Following Angela Creager and Hannah Landecker’s call, this article foregrounds instrumentation and technique in accounting for innovation.^12^ Returning to Merton’s multiples, this focus allows to explain the omnipresence of multiple discoveries or inventions, of which transgenic mice are a prime example. While individual trials, perseverance and tinkering were important, two kinds of infrastructure proved essential, one relatively established (embryological instrumentation, methods, access to mouse houses) and one emerging (techniques and networks of exchange of recombinant DNA molecules). Access to both enabled multiple tinkerers to arrive at a similar outcome from multiple theoretical angles and modes of collaboration.

## ‘Possibilities and realities’

The luminary molecular biologist François Jacob opened his 1977 lecture at the University of California, Berkeley, with a discussion of monsters and their role in delineating the future. ‘Monsters’, he argued, ‘show how a culture handles the possible and marks its limits’.^13^ In a sweeping overview, Jacob touched on the different modes of inquiry and explanation, on a trajectory from magic to science. His main argument was about evolution, that one should best think about natural change as ‘tinkering’ – a concept he adapted from Levi-Strauss and juxtaposed with an engineer’s careful plan:

The action of natural selection has often been compared to that of an engineer. This, however, does not seem to be a suitable comparison. First, because in contrast to what occurs in evolution, the engineer works according to a preconceived plan in that he foresees the product of his efforts. Second, because of the way the engineer works: to make a new product, he has at his disposal both materials specially prepared to that end and machines designed solely for that task. Finally, because the objects produced by the engineer, at least by the good engineer, approach the level of perfection made possible by the technology of the time. In contrast, evolution is far from perfection … it works like a tinkerer who uses everything at his disposal to produce some kind of workable object.^14^

Jacob’s lecture was published in *Science*, and his speculation about evolution happening through major rearrangement of genetic programs at the molecular level has had some influence on evo-devo approaches in the 1990s.^15^ At no point was genetic engineering mentioned, but the omission was rhetorical: the subject was constantly alluded to and flirted with, from the introduction devoted to monsters, to distinctions between engineering and tinkering modes, to a section on ‘molecular tinkering’. When Jacob included this lecture as an essay in his 1982 book, *The Possible and the Actual*, he added a new conclusion explicitly about recombinant DNA, suggesting the public anxieties had proved unjustified, and had stemmed from the fear of hybrid monsters.

Even before Jacob spelled out the relevance of tinkering to genetic engineering, his lecture had been received in that light. At the 1978 Ciba Foundation symposium on ‘Genetics and Human Biology: Possibilities and Realities’ in London, Sydney Brenner’s introductory remarks picked up Jacob’s ideas. Referring to contemporary debates, Brenner argued that genetic engineering in the sense of designing an organism had not been developed. ‘All we can do is a little “tinkering”, but that, as François Jacob … has pointed out is nature’s way and not ours’.^16^ Jacob was in the audience, alongside other luminaries from the fields of molecular biology, developmental biology and genetics, including Walter Bodmer, Francis Crick, Richard Gardner, John Gurdon, Henry Harris, Hilary Koprowski, James Neel, Guido Pontecorvo, Frank Ruddle, and Charles Weissmann. In the speculative atmosphere of the symposium, participants discussed papers on recent advances and possible futures in genetics, from cultural evolution to cancer. Several times, the conversation returned to the possibility of introducing foreign DNA into a mammal.

The idea of introducing genetic changes into animals (including humans) had been at the forefront of ‘new biology’ discussions in the 1960s. Despite the focus on bacteria in the recombinant DNA controversy of the 1970s, the interest in using the new methods to manipulate animals and plants was strong. In the 1970s the ability to manipulate eukaryotic genes in bacterial and eukaryotic cells attracted many scientists to recombinant DNA. By using small circular bacterial DNA molecules, known as plasmids, newly isolated genes could be propagated in bacterial cells, and bacteria could be induced to take up the new plasmids. In the late 1970s, these methods were expanded to cultured animal cells. The molecular promise also captivated a number of laboratories that sought to apply the power of genetics to differentiating embryos. Their programs had often predated the expansion of recombinant DNA tools and relied on the burgeoning research into animal viruses, studies of genes and chromosomes in cells cultured *in vitro* (somatic cell genetics), and nucleic acid biochemistry.

Early attempts to introduce nucleic acids into embryos focused on viruses, reflecting the major US investment in tumor virus research.^17^ In 1974, the first attempt to insert a virus (SV40) into the mouse embryo at the blastocyst stage was published. It was a collaboration between Beatrice Mintz, a mouse developmental biologist at the Fox Chase Institute for Cancer Research, and Rudolf Jaenisch, a postdoctoral molecular biologist at Princeton who specialized in viruses. Using cumbersome techniques to isolate SV40 DNA, Jaenisch would drive to Fox Chase where he would inject the DNA into mouse embryos, under Mintz’s expert guidance. Eventually, they could demonstrate that SV40 DNA would spread dramatically in the embryo, but would not function. While the results suggested incorporation of DNA into the mouse genome, the publication was not framed in these terms. The focus was on whether viral DNA became deactivated during embryonic development, and while the two likely speculated as to the possibilities of genetic modification, they chose to avoid such discussions in the publication.

The controversy over recombinant DNA was just beginning in 1974, with first claims to introducing genes from the *Xenopus* frog into bacterial cells announced the same year. In the following years, a self-imposed moratorium on recombinant research was introduced, and discussions over safety and containment took place at the Asilomar meetings, that divided scientists and fed journalist interest.^18^ In response to the meetings and great public concern as mediated through activism and the press, the US National Institutes of Health (NIH) introduced extensive guidelines on recombinant experiments which, at least in their initial iteration, put stringent restriction on experiments and spelled out extensive requirements for safety procedures and containment facilities. Given the political difficulties around genetic engineering, suggestions for future research tended to no longer be aired in public, and scientific papers were rarely a space for such speculation. Yet writing that addressed expert audiences without a firm commitment to presenting experimental results – review articles, ‘trends’ pieces, lectures and talks – did discuss potential experiments. These sources hint at multiple plans to genetically modify animals in the late 1970s, even though the most promising routes – or species – were by no means settled.

**Figure 1.**
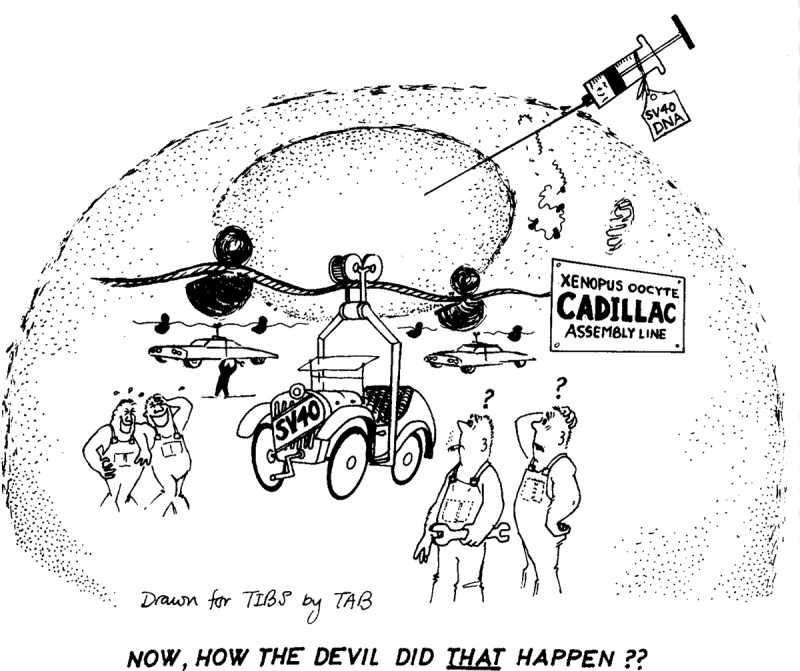
**‘***Xenopus* oocyte Cadillac assembly line.’ A drawing from Gurdon’s programmatic review in *Trends in Biochemistry* showing transcription and translation of injected SV40 DNA on an industrial scale. De Robertis et al., ‘Injected living cells’, 251. Reproduced with permission.

Many experimental organisms, or systems around specific cell types, were promising due to existing work. At the Laboratory for Molecular Biology (LMB) in Cambridge, UK, John Gurdon was turning the frog oocyte – an immature egg cell – into a powerhouse for molecular analysis. Celebrated for cloning *Xenopus* by transferring a nucleus from a tadpole cell into an enucleated egg in 1958, Gurdon articulated the need to understand gene regulation to decipher the mechanisms of development.^19^ With a strong emphasis on molecular thinking as a way to understand eukaryotic biology, Gurdon and colleagues were representing the *Xenopus* oocyte as a simple system that resembled bacteria and could become attractive to molecular biologists. Moving away from the discussions of embryonic complexity and determination common in developmental discourses, Gurdon repeatedly employed the metaphors of a test-tube and a factory to describe the oocyte (Figure 1). In reviews and forward-looking pieces, Gurdon and colleagues argued for the advantages that oocytes offered over cell-free experiments and bacteria, claiming that the ‘crudest’ system such as the living eukaryotic cell might also be best for detailed biochemical analyses.^20^

While Gurdon’s focus may have been on large volume biochemistry, there were speculations that it might lead to introducing genes into *Xenopus*. At the 1978 Ciba meeting mentioned above, lively discussion arose as to which experimental systems would be best suited for such an approach:

*Brenner:* *…* there is also the problem of assessing the value of genes that may have to work in entire organisms to produce their effects. Could you comment on the very long-term idea of putting genes back into organisms?

*Weissmann*: I think that John Gurdon’s system will eventually lend itself to this approach. It should be possible to do the injections [into oocytes] in such a way that one ultimately gets development of the embryo and takes the inserted DNA through the complete cycle.^21^

In the same meeting, Brenner described such idea of putting a gene into a multicellular organism as ‘long-term’, but others disagreed. Many even suggested that mammals might be the key focus for genetic modification. The key hopes were still linked to cultured cells, especially teratocarcinoma lines – cells derived from mouse teratoma cancers that showed properties of embryonic cells (these were eventually supplanted by embryonic stem cells in the 1980s). Beatrice Mintz’s laboratory was one place heavily invested in teratocarcinoma cells. Mintz hoped that once a mouse could be produced from the teratocarcinoma line, ‘the animal would literally be the model of the human disease’, but her lab was still having problems making teratocarcinomas contribute to the germline, which would allow for future generations of mice derived from these cells.^22^ If the questions among the geneticists at the Ciba meeting focused on the way to trace a gene through the developing embryo, Mintz was concerned much more with development and cancer, and understanding malignancy in developmental terms: whether tumors arose from one cell or many, which factors affect their growth and differentiation, and whether the key changes were genetic.^23^ To this end, she hoped to use genes as markers to track differentiation rather than a primary subject of investigation, by looking for new informative mutations:

Through mutagenesis and selection *in vitro*, followed by differentiation *in vivo*, experimentally useful genes could be introduced into mice. There, the full developmental consequences of specific gene mutations coding for biochemically identified changes could be brought to light.^24^

Teratocarcinoma cells seemed the perfect system for such work, as they offered both the possibility to be modified in vitro, and to be introduced into mice. At the 1977 Brookhaven meeting on gene transfer, Mintz made the point even more strongly: she referred to teratocarcinomas as ‘surrogate eggs’, cells that could integrate the whole-organism approach of classical genetics with the selection advantages offered by somatic cell genetics. She specifically discouraged the use of mouse ova, as these were too few in number and impossible to select in culture, while other kinds of somatic cells could not contribute to the embryo since they were already differentiated and their genetic status was ‘usually uncertain’.^25^ By contrast, teratocarcinoma cells had the potential to fulfil both criteria, even though their genetic status, as cancerous cells that accumulated mutations, was not exactly certain, either.

Teratocarcinomas as a vehicle for genetic modification were also being explored by Karl Illmensee, who moved out of Mintz’s lab to work with Carlo Croce at the Wistar and subsequently secured a permanent position at the University of Geneva in 1977. Today, Illmensee is associated with the allegations of fraud surrounding his claims to have cloned three mice in 1981, but this controversy did not break until 1983.^26^ In the late 1970s, he was a rising star in developmental biology, garnering a reputation as a virtuoso experimentalist and a charismatic public speaker. With Croce, Illmensee hybridized teratocarcinoma cells with different somatic lines, hoping to make a hybrid that would contribute to the developing embryo and perhaps introduce novel mutations from somatic cell lines into the whole mouse. Moreover, Illmensee established a collaboration with Peter Hoppe, a mouse embryologist at the Jackson lab, with whom he attempted making parthenogenetic eggs (eggs induced to begin development without fertilization). After a series of high-profile publications, Illmensee was writing programmatic review papers and gave several keynote lectures in 1980.^27^ These were devoted to genetic modification of the embryo, and the methods he proposed were diverse, covering his cell hybrid work, parthenogenesis and nuclear transfer, and differed significantly from those that proved successful.

In these discussions, recombinant DNA was not the primary tool for the job, nor had it always been entertained as relevant. Recombinant techniques were still novel and inaccessible to most developmental biologists, even they were followed with interest as a promising new tool. A review in *Developmental Biology* published in 1979 gives a good idea of envisioned applications.^28^ The focus was decidedly on the new information about eukaryotic genomes and on isolating specific genes known to be involved in development. The review highlighted gene regulation and its possible role in differentiation – a well-established molecular take on development by then – as well as the two gene families that were receiving much attention for their roles in disease and immune response: globins (components of hemoglobin and similar proteins) and immunoglobulins (antibodies). Even in its more speculative parts, however, the review did not raise the prospect of introducing isolated genes into the embryo.

The most obvious direction, then, seemed to be about interrogating eukaryotic gene regulation in simpler systems: cultured cells or even as isolated molecules *in vitro* – practices much more consistent with the triumphant model of microbial molecular biology. As Tim Stewart, Mintz’s postdoc who ended up succeeding at injecting DNA into mouse eggs, pointed out,

Most of the DNA … transfectional work going on … was primarily focused on understanding the relationship between gene structure and expression, as opposed to: what is the gene product doing in the context of the whole animal? And so when you think about, if that’s your motivation, trying to understand gene expression, [introducing genes into animals] seemed like a hell of a lot of work for pretty modest pay-offs.^29^

Despite conflicting ideas as to what genetic manipulation of the mammalian embryo might look like, several laboratories simultaneously pursued experiments that involved microinjecting DNA into the mouse egg. Discussions of possible uses crystallized around existing laboratory practices and agendas of individual researchers to become biological reality. Significantly, they drew on the growing infrastructure of gene exchange, as well as established embryological techniques, but all of this work involved extensive trial and error, and mastering new tools – practices that are best described as tinkering.

## Research agendas

**Figure 2.**
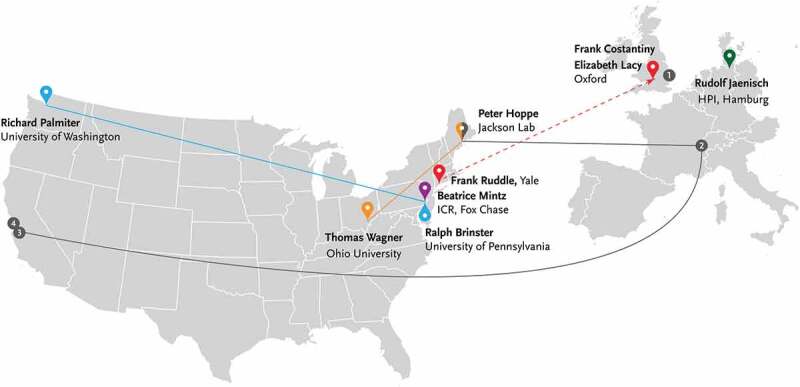
Laboratories that worked on microinjecting DNA into fertilized mouse eggs in 1979–81. Solid lines indicate collaboration, dashed line represents teaching of techniques. Numbers on the map: (1) Keith Willison in John Gurdon’s laboratory, MRC Laboratory of Molecular Biology, Cambridge; (2) Karl Illmensee, University of Geneva; (3) Axel Ullrich, Genentech; (4) Howard Goodman, University of Caifornia, San Francisco. ICR isInstitute for Cancer Research, HPI – Heinrich-Pette Institute.

Genetic modification of a whole mouse, while entertained in increasing detail, was not a clearly articulated agenda in 1980, and many prominent biologists relegated it into the realm of distant possibilities. However, in 1979–80, at least eight groups worked on introducing various forms of DNA into cultured mouse embryos at the one or two-cell stage, with six of them eventually reporting successful results. As I will show, these were mostly speculative projects pursued by postdocs or graduate students in well-funded laboratories that were integrating new molecular tools into their everyday practice. Most of these had secure funding, a certain level of prestige, and access to the fledging exchange networks of plasmids with isolated genes. These laboratories, mapped in Figure 2, pursued experimental programs over a considerable disciplinary range.

Overall, they represented two important trends in 1970s biology: the growing interest of molecular biologists in multicellular organisms, and developmental biologists’ engagement with molecular tools and models.^30^ The NIH relaxed its recombinant DNA regulations in 1978 and permitted wide-ranging work with eukaryotic DNA and cells, with even further deregulation in 1980. Yet while the promise of genetic modification of mammals was being discussed, converting this into a research agenda was not straightforward. Various audiences had to be convinced that these experiments were worthwhile and fundable pursuits. These projects therefore tended to be spin-offs from already established grants – a practice known as ‘bootlegging’.^31^ Thus, Ralph Brinster at the University of Pennsylvania deemed DNA injections into mouse embryos ‘unfundable’, and instead built his successful grant applications to both the NIH and the National Science Foundation around RNA injection in the mouse, inspired by John Gurdon’s work in the frog.^32^ These RNA grants then bankrolled the DNA injection work. In Beatrice Mintz’s lab, the DNA injection span off the teratocarcinoma project. At Yale, Frank Ruddle relied on his generous somatic cell genetics grant from the National Institute of General Medical Sciences to assign a graduate student and then a postdoc to the project.^33^ Overall, the scientists I discuss here relied on several kinds of infrastructure to pursue this speculative work: access to flexible funds, access to embryological equipment, and participation in the new informal networks of gene exchange, before the standardization and widely available expertise in gene manipulation.

Gurdon’s experiments on nucleic acid injections in *Xenopus* were an inspiration for mouse embryologists beyond his group. Since 1979, Brinster’s laboratory in the University of Pennsylvania Veterinary School in West Philadelphia was abuzz with work on manipulating mouse eggs, including injecting RNA and DNA molecules. Like Mintz, Brinster had adopted microinjection in the early 1970s to introduce cells into blastocysts, another side-project that he funded by using his embryo culture grants to purchase microinjection equipment.^34^ With postdocs Mary Avarbock, Howard Chen and Myrna Trumbauer, he developed the technique to work with the even smaller mouse eggs. While Brinster was an experienced embryologist with the capacity for honing micromanipulation techniques in his own laboratory, the purified nucleic acids came mostly from Gurdon’s collaborators.^35^ In autumn 1979, Brinster contacted Richard Palmiter, a biochemist at the University of Washington in Seattle, to secure ovalbumin RNA. This connection resulted in a long-term collaboration, in which Palmiter supplied plasmids for the future gene transfer work.^36^ Brinster’s institutional position at a major university enabled him to benefit from the networks of exchange with molecular biologists, who had provided samples of nucleic acids that could be expanded into new systems. In January 1980, Brinster published on a successful protein synthesis of rabbit beta globin from RNA in mouse eggs, while his group was working on injecting DNA and trying to trace its presence in adult mice at the same time.^37^

Only a short train ride from the University of Pennsylvania, in the suburb of Fox Chase, Mintz’s group carried on working with teratocarcinoma cells, although not in communication with Brinster. In 1979, Mintz started a collaboration with Richard Axel’s laboratory at Columbia.^38^ Their work brought together expertise in culturing teratocarcinomas and gene transfer into mammalian cells. Using a teratocarcinoma mutant line that lacked functional thymidine kinase, the researchers inserted a plasmid containing the HSV *tk* gene combined with the human beta globin gene that had been purified by Maniatis, Axel’s collaborator and friend. The plasmid remained in Mintz’s laboratory and was used in new experiments.

Quite how much anyone knew about what was going on in Mintz’s laboratory is an open question. Her teratocarcinoma research certainly put the lab on the radar as a place where a genetically modified animal could soon be born. Her postdocs’ recollections refer to multiple conversations about possibilities and research plans.^39^ Apart from the sense of expectation from scientists in other labs that they recalled in interviews, this is evidenced by a letter from Paul Berg to the RAC that inquired about whole animals as hosts for recombinant experiments – something that had seemed like a fantastic possibility only a few years earlier. In the letter, Berg stressed the ‘tremendous scientific and medical importance of such experiments’, and wrote:

As you know the experimental ground work for [introducing recombinant DNA into whole animals] has already been provided by Dr. Beatrice Mintz’s experiments. She has shown that teratocarcinoma cells grown in culture can be incorporated into mouse blastocysts which ultimately can yield mice containing a variety of cell types derived from the teratocarcinoma cells. Using appropriate selections it is feasible to introduce exogenous DNAs (be they derived by recombinant techniques or otherwise) into such teratocarcinoma cells and hence into animals.^40^

Mintz’s former collaborator Rudolf Jaenisch continued to work with viruses. After spending five years at the Salk Institute, he was recruited to direct the Tumor Virology department at the Heinrich Pette Institute, University of Hamburg, in 1977. There, he spent several years setting up a mouse facility and continued working on introducing viral DNA into the embryo. Using the mouse Moloney leukemia virus, Jaenisch and his colleagues injected it into embryos at various stages: blastocyst, four-cell embryo and eventually fertilized one-cell egg. Their questions centered on preferential viral gene expression in specific tissues and the effect of an embryonic environment on viral replication and activity. Yet, despite the great investment in animal virus research, with the expansion of recombinant DNA, viruses were becoming less attractive as a means of delivering genetic information into cells.^41^

It was not just developmental biologists who pursued genetic modification of the mouse embryo. Two final research programs I will discuss came from less expected trajectories. One was Frank Ruddle’s lab at Yale with a catholic focus on somatic cell genetics. The other was the result of a partnership between Peter Hoppe – a mouse embryologist at the Jackson Lab and Illmensee’s collaborator – and Thomas Wagner, a biochemist at Ohio University.

Ruddle’s approach to somatic cell genetics went beyond traditional disciplinary divides that were increasingly irrelevant in the world of biomedicine. He ran a large laboratory of about 30 people and made efforts to connect to diverse communities of practicing biologists. Ruddle’s extensive participation in academic networks combined with his secure institutional position allowed him to start multiple collaborations, and his eclectic approach brought many techniques to his group. Ruddle’s laboratory was exploring chromosome-mediated gene transfer – effectively shifting chromosomes between cells – and in pursuit of higher efficiency, he sought new means of incorporating new genes into somatic cells. Learning of microinjection from Elaine Diacumakos at Rockefeller University, who had worked extensively on honing the technique, Ruddle eagerly adopted the apparatus and introduced a microinjection room in his laboratory, with an NSF instrumentation grant and help from Yale developmental biologist Clement Markert.^42^

Despite multiple scientific commitments and co-authored papers, Ruddle’s main focus was on mapping human genes in somatic cells. Experiments with recombinant plasmids that he labelled DNA-mediated gene transfer offered an even higher degree of resolution, as transferred genes could be radioactively detected on specific parts of the chromosomes. The injection of DNA into embryos, on the other hand, was a speculative experiment that Ruddle hoped could be useful for developmental studies. He applied to inject recombinant DNA into mouse embryos to Yale’s Biological Safety Advisory Committee and received approval in March 1979.^43^ Initially, Ruddle assigned a graduate student to the project, with little success, and then decided to recruit a postdoc.^44^ This was Jon Gordon,^45^ who had just completed a thesis with Clement Markert at Yale, work for which involved making chimeric mice. With much experience in micromanipulation and dealing with mice, Gordon was assigned to the embryo project and given considerable autonomy.

The final group that worked with mouse eggs was the collaboration between Thomas Wagner at Ohio University and Peter Hoppe, a Jackson Lab-based developmental biologist. Wagner was a molecular biologist with a background in the physical chemistry of DNA who had set up Ohio’s Department of Molecular and Cell Biology in 1970. Wagner struggled to secure large-scale federal funding for his department, and despite the administration’s interest in building up its research programme, the university was at the periphery of molecular research. Wagner’s experimental focus was largely structural, but through contact with Azim Surani, a developmental biologist at the Animal Research Station in Cambridge, UK, Wagner became interested in how DNA was regulated during development. In the late 1970s, he worked on mouse sperm DNA, and published a series of papers on its structure. The sperm nucleus is extremely small and it had been recognised that sperm DNA was packaged much more tightly compared to other cells, and that it had a different kind of protein in its chromatin. Through structural calculations and experimental analysis of sperm DNA, Wagner hypothesised that it had to contain breaks to fit the sperm nucleus.

The notion of broken-up sperm DNA was unorthodox, and Wagner published his results in *Archives of Andrology*, unlikely to be read by molecular biologists.^46^ However, for Wagner, these results implied that sperm DNA had to undergo a series of repairs after fertilization. At this point, he suggested, it might be possible to introduce foreign DNA into the host genome. Wagner shared his thoughts with Hoppe on a taxi ride to the airport at a conference in Washington, D. C. By then, Hoppe had already been collaborating with Illmensee, and said he could carry out an injection into the male pronucleus. On his return, Wagner sent a rabbit beta globin plasmid that he had obtained from Richard Flavell in London via a postdoc and sent it to Jackson Lab for injection.^47^

Not all experiments took place in established mouse labs. John Gurdon’s injection work on *Xenopus* continued at the Laboratory of Molecular Biology (LMB) in Cambridge, UK, and one of his PhD students, Keith Willison, decided to pursue mouse embryos. Willison attempted to inject SV40 DNA into two-cell embryos and into blastocysts, with some results suggesting he could detect injected DNA in adult mice. Expertise in manipulating mouse embryos came from elsewhere: the Cambridge Anatomy and Genetics departments (notably from Martin Johnson and Martin Evans), as well as Willison’s relationship with Oxford embryologists. The overall inspiration and guidance came from Gurdon, but Willison obtained SV40 DNA and learned the latest methods from Janet Mertz, who had moved to the LMB from Stanford. Mertz had been a graduate student of Paul Berg – indeed, it was her proposed experiments with SV40 genes that had started the recombinant DNA controversy in 1973.^48^

Towards the end of his PhD, an institutional contingency severely damaged Willison’s research; with no mouse facility at the LMB, he kept his animals at the Addenbrooke’s Hospital, where the whole colony was culled without any warning after a viral infection. Salvaging some results that he had collected, Willison submitted his thesis in late 1978, with final corrections approved in December 1979. By then, he had moved on to a postdoc at Cold Spring Harbor to learn the recombinant techniques and had set his mouse work aside. Rudolf Jaenisch cited his thesis in a 1981 paper but overall it was not easily available and any hints of results would only spread through word of mouth.^49^ While the culling of Willison’s mice was unpredictable and unfortunate, it highlights the importance of solid infrastructure for speculative work. An institutional commitment to using the mouse as a laboratory organism, combined with local expertise from animal technicians and scientists was common to those laboratories that ended up publishing such experiments.

The key participants above largely agree that their work was independent. A few expressed their suspicions about premature familiarity with unpublished data in interviews, but quickly pointed out they had no certain knowledge either way. The exact chronology of these projects thus poses a challenge. While the record of publications is clear, it is by no means a straightforward way to establish the timing of the work. Without access to laboratory notebooks, it can only be extrapolated from ephemeral and circumstantial evidence. Ruddle initiated the experiment after the NIH guidelines were relaxed in December 1978, and commented on the preliminary results to *Yale Daily News* in April 1979, and had his research program clearly advertised in the 1980 issue of *General Embryological Information Service*, a developmental biology newsletter.^50^ However, not all groups were as open about their research in progress. Recruitment of postdocs who performed the experimental work at Yale and Fox Chase offers another bookend: most of them joined in 1978–79. The sequence of Fox Chase scientific reports shows that Mintz had not listed the egg project as of September 1980, and whether it had been attempted before is unclear. Brinster reported some preliminary success with DNA injection in a note added in proof to one of his RNA papers that appeared in print in January 1981, a few months after Ruddle announced his group’s success.^51^

A few other attempts that had not led to publication can also be traced. Howard Goodman – famous for a conflict with Genentech over work on the insulin gene – applied to RAC in early 1980 to insert rat insulin DNA into a two-cell mouse embryo, even though no results came out of this project.^52^ Illmensee attempted to inject a plasmid containing the mouse beta globin gene into a mouse egg, in an experiment designed to distinguish between the native and foreign versions of the protein. However, such work was seen as a long shot, the protein from the plasmid could not be detected and the work was therefore never published, surviving only as a ‘personal communication’ in Willison’s PhD thesis.^53^

It is worth remembering that uncertainty about which exact experiments were in progress, and where, was a condition that the scientists faced, too. The idea that such work was happening elsewhere, while the credit was yet to be allocated, was a motivation to go forward, and created a sense of competition. Rumors circulated about what other labs were doing. As Tim Stewart, then Mintz’s postdoc, noted, ‘it’s always been very hard to keep secrets, people talk about what they’re doing, people are excited about what they’re doing and they don’t want to keep it a secret’.^54^ On the other hand, no officially published claims had been made and the experiments remained a prize worth pursuing, even if the risks were high for junior scientists. The experimental procedures were time-consuming and uncertain to yield results, and therefore publications, but they responded to a partially articulated idea of a genetically modified mouse.

## Tinkering with microinjection

The emergence of developmental biology as a coherent identity has been associated with the expansion of communication between embryologists, geneticists and molecular biologists at conferences and new journals, and cemented in textbooks and undergraduate courses.^55^ But synthesis of experimental practices was less straightforward. In a few labs, a hybrid way of practicing developmental biology emerged in the 1960s and 1970s. The most famous hybrid practices were devised by molecular biologists moving to work with embryos, such as Sydney Brenner with *C. elegans*, François Jacob with mouse teratocarcinoma cells. John Gurdon with *Xenopus*.^56^ However, such synthetic approaches to what Gurdon called ‘molecular embryology’ were not the rule. In all laboratories that worked on gene transfer into mouse embryos, there was a division of labor between molecular biologists and embryologists. The elaborate techniques of microinjection became a crucial embryological intervention these groups had to master. These techniques, drawing on the instrumental tradition of embryology that involved crafting tools and embodied skills of manipulation, were a prime example of tinkering.

Hannah Landecker has traced the construction of animal and plant cells as technologies in twentieth century biology, through cell culture and associated techniques.^57^ Microinjection was part of this trajectory. A form of microsurgery or ‘micrurgy’, it was a practical approach established in biological research since the turn of the twentieth century. First widely employed in bacteriology to isolate single cells, microsurgical procedures relied on micromanipulators – instruments that converted the manipulation of screws, knobs or joy-stick-style controls into microscopic three-dimensional movements of capillary needles or pipettes.^58^ The first commercially available micromanipulators appeared in the 1930s; by the 1960s Leitz models dominated the market.^59^ If much early microscopic work in experimental embryology had been done by directly holding instruments, nuclear transfer and chimera experiments relied on motion aided by a micromanipulator.^60^ Scientists at the key sites of making chimeric mice were adept at microinjecting blastocysts: Mintz’s and Brinster’s laboratories had integrated the technique into their everyday practices. The craft-like nature of micromanipulation was stressed routinely in contemporary accounts and in recollections. Thus, Karl Illmensee’s remarkable experiments and his growing reputation in developmental circles partly relied on his perceived ‘golden hands’.^61^

**Figure 3.**
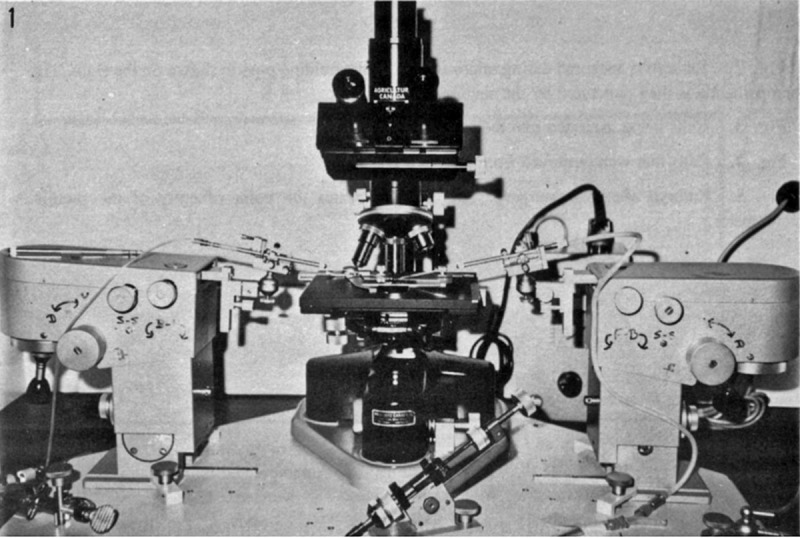
A typical embryology microinjection set-up, showing two Leitz micromanipulators and a microscope. From Betteridge, Hare & Singh, ‘Approaches to Sex Selection,’ 117. Reproduced with permission.

Beyond the embryological tradition, micromanipulation was used by cell biologists, mostly to study the effects of removing specific organelles and even chromosomes. Since the 1960s, Elaine Diacumakos at Rockefeller University and Adolf Graessman’s group at the Free University in West Berlin pursued the technique with limited communication beyond their immediate colleagues. Through growing interest in cell manipulation – encouraged by the expansion of somatic cell genetics – these techniques attracted several geneticists. Thus, both Ruddle and gene therapy pioneer French Anderson approached Diacumakos to learn her methods, and microinjection became a potential tool for gene transfer into eukaryotic cells in the late 1970s (Figure 3).^62^

Microinjection experts promoted their techniques from two angles, that invoked distinct images of the cell. On the one hand, they highlighted the simplicity and utility of these tools for a broad range of biologists, arguing that the procedure could turn a cell into a test-tube. The cell-as-test-tube metaphor was used in reviews from the Gurdon and Graessman groups.^63^ As Hannah Landecker argues, cell fusion experiments and micromanipulation in the 1970s came to offer the legitimacy of working in a living environment of the cell, while also removing barriers of species and immunological incompatibility in making hybrids, allowing much more ambitious experiments.^64^

On the other hand, micromanipulation pioneers also extolled the virtues of working with ‘natural’ cells and implied that their method could finally conquer eukaryotic complexity.^65^ Thus, addressing a mixed scientific audience interested in gene transfer, Diacumakos expanded the test-tube metaphor and suggested that:

Another disadvantage [of microinjection], which is only superficial, is that ‘quick and dirty experiments’ are not doable. The design of experiments using this approach must therefore be defined and clear-cut … This is not the type of activity that someone with no knowledge of cell biology can perform and interpret accurately … [T]his is an approach that should be considered seriously, for the cell is not merely a test tube; in the hands of a good cell biologist, the cell itself can become a laboratory.^66^

Embryos and ova were part of similar ambitions. In the labs that decided to microinject plasmid DNA into mouse eggs in-house, this practical expertise was carried by the postdocs who performed the experiments. The experimental design appeared straightforward enough: DNA was to be injected into a pronucleus, with the egg subsequently implanted into the oviduct of a surrogate mouse. But attempting such a speculative project was a career risk. As postdocs without secure prospects, despite being placed in highly-respected laboratories, they required publications of impressive results to secure a permanent position. As Jon Gordon from the Yale group recalled,

In Frank [Ruddle]’s lab, the atmosphere was that this was more or less a speculative effort. A lot of big-time labs were unable to do it, I mean, Beatrice Mintz, it’s a big time lab … so I don’t think a lot of people felt that it was very likely I would do it, and I told Frank that, I said ‘Hey, look, I need a back-up project. I’m on a postdoc here, what if this isn’t possible? Can I map a gene?’^67^

Similarly, Tim Stewart (Fox Chase) said:

When I got there I knew that Bea [Mintz] had been writing grants, talking about this. There was a lot of skepticism and I remember talking to one of the postdocs at Fox Chase and I think there might have been skepticism outside the institution, can you actually do it? – and even if you could, how much value are you *really* going to get out of this? […] getting [DNA] into an embryo and then having that embryo survive the trauma of this manipulation and to get a whole animal out of it was considered a long shot.^68^

The decision to work with one-cell fertilized eggs was not obvious. The mouse egg had the reputation of being difficult, by contrast with the rabbit embryo, a more ‘robust’ subject of manipulation. Similarly, the optimal developmental stage at which to inject remained unclear. John Gurdon relied on the frog oocyte – a precursor of the mature ovum – as more reliable, whereas the freshly fertilized frog egg was seen as extremely fragile until it approached its first division.^69^ Diacumakos may have stressed that the nucleus of a somatic cell was much more robust than previously thought, and able to withstand injection, but it was a different matter to make the experiments work in another lab and with a different type of cell.^70^

Moreover, for mammalian development, the ‘two-cell block’ was an important concern: whereas eggs at the two-cell stage could be forced to develop to blastocyst and then implanted into the uterus, eggs extracted just after fertilization would only divide once *in vitro* and degrade. Methods had been developed to overcome the two-cell block, by adding oviduct extract or otherwise changing the culture medium.^71^ Still, the phenomenon itself remained mysterious, with suggestions that it was affected by the strain of mice used. Ways of avoiding the two-cell block had not been routine, since most embryological experiments did not require them. This concern about the two-cell block led Keith Willison to inject mouse eggs at the two-cell stage; Jon Gordon at Yale also attempted two-cell injections, with little success.

**Figure 4.**
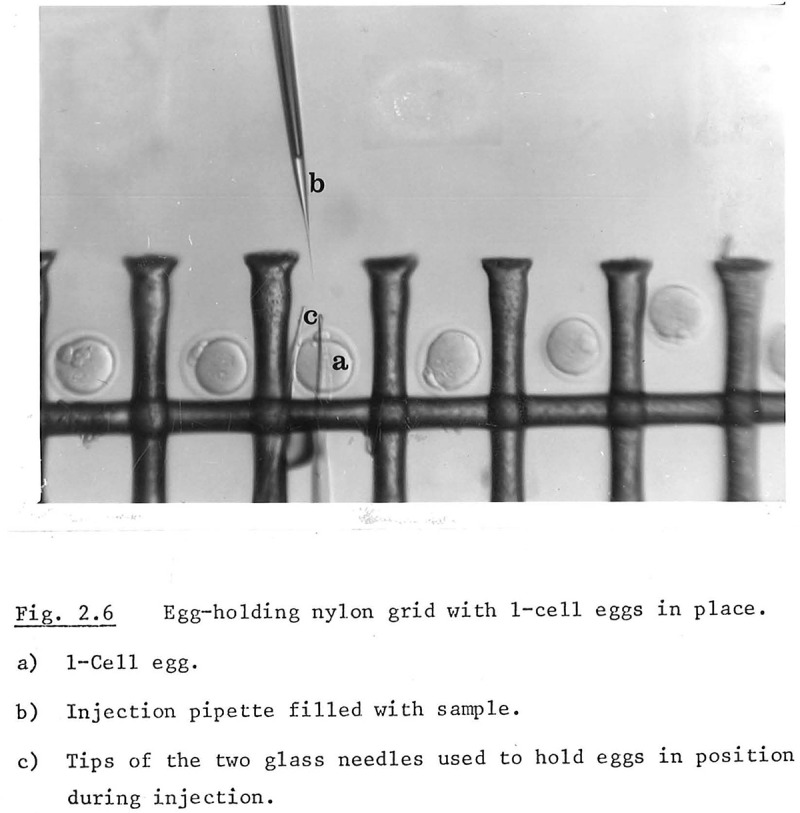
Keith Willison’s unusual microinjection set-up, which involved a nylon grid to hold embryos in place. While one-cell eggs are used in the image, their manipulation did not make it into the thesis text. From Willison, *Microinjection of Mammalian Eggs*, 24, Reproduced with permission.

Existing techniques were being reinvented from scratch, and many avenues led nowhere. Part of the problem was that the criteria of a successful experiment were by no means agreed, because nobody expected that most injected eggs would survive the process and have detectable DNA. Contamination could not be ruled out straightforwardly. Issues of efficiency were also at stake because selective detection of successfully modified embryos was not an option, in contrast to cell culture. Multiple eggs had to be injected in one go, which required making the procedure routine and keeping the timeframe of embryo transfer as brief as possible to avoid the two-cell block – a technique Gordon and Stewart both settled on independently. They cultured multiple eggs and injected them in series, sucking each egg with a holding pipette. Willison came up with a different solution, adapting a Nylon grid to keep the eggs in order – a method that never spread, probably because it remained in a PhD thesis (Figure 4).

Microinjection was a fraught procedure that relied on embodied skill and ad hoc solutions. Adapting local instruments, developing ways of focusing the optics and arranging the pipettes to achieve best results, as well as training the eye to tell when a pronucleus was pierced and successfully filled with the DNA solution all took time and effort, and the skill set was not easily portable. It is uncertain how many others attempted similar experiments. However, success in the enterprise did not rely solely on injection skills or the perseverance of dedicated postdocs working long hours. It was also crucial that experimenters had access to DNA to inject as well as means of tracing it once the embryos were transferred into surrogate mothers. This relied on a synthesis of molecular and developmental practices, for which there were many possible arrangements.

## Recruiting molecular expertise

Many scientists were thinking about development in molecular terms and envisioning experiments that could help interpret gene expression, but combining molecular and developmental practices was difficult. While some molecular-oriented laboratories had embraced a synthesis of both approaches in the late 1960s, there was still a sharp division of labor and identities, even in spaces that encouraged interdisciplinary research. The laboratories that developed microinjection into mouse eggs combined these kinds of expertise in several distinct ways, which were crucial to their success. In the process, they also relied on an emerging infrastructure of gene exchange, which enabled these kinds of experiments.

Getting hold of genes that could be injected and then detected in the mouse embryo was not straightforward in 1980. A lab either had to have a recombinant DNA research programme in its vicinity, furnished with appropriate containment facilities as regulated by the NIH guidelines or their equivalents abroad, or it had to access a gene exchange network.^72^

**Figure 5.**
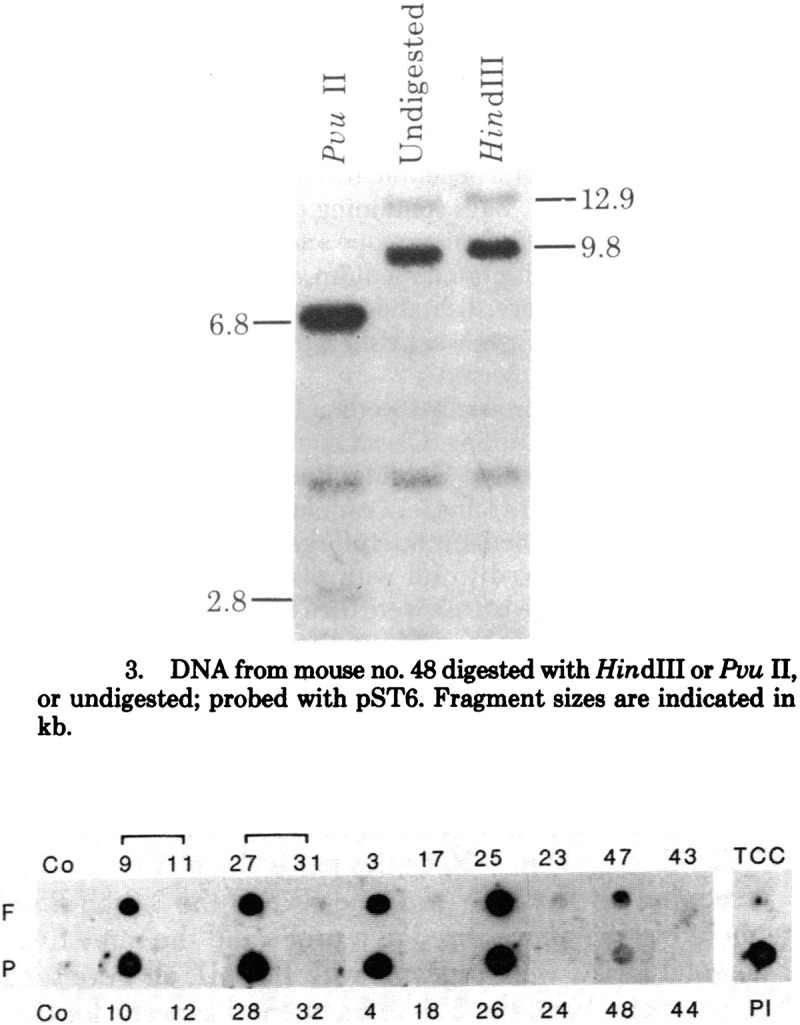
Visualising DNA. *Above*, Southern blot image from Gordon and Scangos’ analysis of the mouse 48 that carried the injected DNA after birth. Below, Tim Stewart and Erwin Wagner’s dot-blot hybrids for fetuses (F) and placentas (P) (their Southern blots were also published). From Gordon et al, ‘Genetic Transformation of Mouse Embryos’, 7382; E. Wagner, Stewart & Mintz, ‘The human beta-globin gene,’ 5017. Reproduced with permission.

Once the DNA was injected, it had to be followed through the embryo and adult mouse, and tested for functionality. These techniques were increasingly routine in molecular laboratories, but not yet standardized or widely disseminated beyond the core molecular community. Finally, to be certain of the potential positive results, proper methods of visualizing and publishing the data had to be followed.

For Mintz and Ruddle, access to plasmids was straightforward. Both had collaborated with Richard Axel and Saul Silverstein at Columbia, the pioneers of injecting DNA into animal somatic cells – Mintz working on teratocarcinomas, Ruddle on chromosome-mediated gene transfer.^73^ Mintz’s laboratory used the dual HSV *tk/*β-globin construct. In Ruddle’s laboratory, Diane Plotkin and Jim Barbosa were working on culturing and modifying the Columbia HSV *tk* plasmid. Moreover, Ruddle’s Yale colleague William Summers had established tests for the expression of HSV *tk*. The virus gene had been used widely in selection experiments, while the globin genes were among the first to be cloned for their relevance to heritable blood diseases. These DNA constructs were not chosen for their value in answering developmental or genetic questions, but simply because they were available and readily detectable.

Brinster had been securing DNAs and RNAs from a variety of high-profile American scientists for his experiments, from Donald Brown to Paul Berg. As his programme of microinjection was expanding, he first collaborated with Carlo Croce at the Wistar Institute around the corner from his laboratory, and eventually settled on a long-term partnership with Richard Palmiter at the University of Washington in Seattle, who had been sending him ovalbumin, metallothionein and HSV *tk* DNA. Even though Palmiter had to go to Strasbourg to perform his first recombinant experiments with ovalbumin due to the lack of proper facilities, by 1980 the University of Washington had built approptiate containment facilities and Palmiter could produce enough DNA to ship from Seattle to Philadelphia.

For Thomas Wagner at Ohio University, access to a plasmid was less straightforward. His laboratory was not at the time pursuing gene splicing, and plasmids were not easy to obtain on the margins of the East and West Coast exchange networks. Luckily, one of Wagner’s students, Christine Schumacher, was moving on to a postdoc with Richard Flavell at the National Institute for Medical Research in the London suburb of Mill Hill. Flavell was one of the first biologists to clone the rabbit β-globin gene, which circulated alongside Tom Maniatis’s construct. On Wagner’s request, Christine Schumacher sent the DNA to Ohio in the post, and Wagner started a collaboration with Joseph Jollick, a biochemist at Ohio University with expertise in recombinant methods.

Analysis of the experiments required a more committed interaction. Collaborations that combined expertise of different laboratories were a common strategy, but both Ruddle and Mintz sought to expand on using molecular techniques that aligned with their research agendas. As primary investigators in highly regarded laboratories, the most straightforward way to gain practical know-how was to recruit expert associates. In 1979, Ruddle hired George Scangos, a microbiologist trained in *E. coli* genetics at the University of Massachusetts. In Ruddle’s lab, Scangos worked on DNA-mediated gene transfer into somatic cells, as well as doing some work on analyzing the molecules in experiments. In June 1979, Erwin Wagner (no relation of Thomas Wagner) joined Mintz’s lab. During his doctoral work on virus genetics in Munich, he had visited Francois Jacob’s lab at the Institut Pasteur were he encountered teratocarcinomas. As Wagner recalls, a division of labor was imposed in Mintz’s laboratory, and his collaboration with Stewart (and learning the associated embryological techniques) had to happen on the side.^74^

By contrast, both Brinster and Thomas Wagner established long-distance collaborations. Wagner worked with Jollick on molecular analyses and immunological tests in attempts to detect rabbit beta globin, relying on Peter Hoppe at the Jackson Lab for embryological manipulations. Brinster’s collaboration was more long-term: Palmiter was attracted by the new project and once the results turned promising, abandoned most of his earlier work on ovalbumin. In 1981, a two-way postal relationship emerged between Seattle and Philadelphia. Brinster would receive the DNA, inject it in his laboratory and send the samples back to Seattle for analysis – an arrangement that persisted throughout the 1980s and was made routine. Coordinating the molecular and developmental aspects of the work was carried out in regular Saturday phone conversations. In fact, Brinster and Palmiter had not met in person until just before the publication of their first paper in November 1981. Once the relationship was stable, Brinster’s lab would conduct the injections, take liver, spleen or tail samples from the resulting mice and send them to Seattle at room temperature in SDS, a chemical that denatured the tissues during shipment and prepared them for analysis. In a sense, FedEx became an extension of their laboratories.^75^

The first products of these analyses were usually Southern or dot-hybridisation blots (Figure 5). The former allowed to determine the size of cut DNA fragments to further confirm identity, the latter gave a yes-or-no answer to the presence of the tested DNA. Since their early dissemination, Southern blots had become the standard in detecting specific DNA.^76^ These images were also the first indication that microinjection worked and the foreign genes could be detected in the newborn mice. They did not provide any certainty as to whether the genes integrated into the mouse genome or persisted as extra-chromosomal elements, but could be straightforwardly applied to a large number of samples, in line with overcoming the problems of efficiency in microinjection. Scangos and Gordon examined 78 newborn mice, born after several hundred embryos had been injected and transplanted into surrogate mothers. The newborn mice were killed and homogenised and the resulting blended mix analyzed. Of these, only two (nos. 48 and 73) showed the foreign HSV *tk* sequences on Southern blots – luckily, since after a beer to celebrate the first positive result, Ruddle sent the pair to look at more blots to see if they could confirm the result. Similarly, Stewart and Erwin Wagner analyzed 33 surviving fetuses, of which five responded positively to the test.

In the accounts of the technique and actors’ recollections about this work, microinjection received the greatest focus as the difficult technique that had to be figured out. By contrast, molecular methods are often taken for granted and were seen as straightforward. Southern blotting, DNA cloning and purification were becoming common, even if the techniques had not been codified yet. These molecular tools became routine during the 1980s, while microinjection remained a laborious process. Yet much local variation and personal knowledge were involved in making molecular methods work, too. Even the most routine methods such as the plasmid prep (a protocol to purify a plasmid) involved local tinkering depending on equipment, training and personal preference.^77^ But the emphasis on the difficulty of the embryo remained an important story in the field, one that had to be actively challenged as transgenic mice propagated in the 1980s.

## Conclusion

A genetically modified mouse had been envisioned and discussed for a long time, drawing on both existing experiments such as the Jaenisch-Mintz work, and applications of new techniques. Heterogeneous approaches were pursued to integrate foreign genes into an animal, in laboratories on a broad disciplinary spectrum. The early success with microinjection happened in the few laboratories that combined specific molecular and developmental expertise, had the infrastructure to support large-scale mouse work and sufficient financial means to pursue a highly speculative and potentially ‘unfundable’ endeavor. Multiple divisions of labor – between molecular and embryological skills, between principle investigators and postdocs, between geographically separated groups – were a common requirement for success. Through the process, scientists relied on available instruments and novel tools and samples, but they tinkered with embryos and genes, devising new methods of handling and manipulating embryos and DNA, relying on resources that were available, and adapting existing tools for new problems.

Distance was an important feature in the overall pattern of multiple invention. As Mario Biagioli points out in his study of Galileo’s strategies of securing credit, knowledge at large is ‘constituted through a range of distance-based partial perceptions’.^78^ In this case, the limited communication between laboratories, the ‘grapevine’ and speculations about what was going on in competing laboratories sustained an interest in pursuing gene transfer into embryos as a promising and cutting-edge line of research. These laboratories avoided the synthetic programs focusing on compromise experimental objects, for instance the ultimately unproductive teratocarcinoma cells. By maintaining the division of molecular and embryological labor in the early day, either within the same laboratory or through postal contact, local problems and minute details involved in manipulation or DNA analysis could be resolved by the expert, whereas material objects were produced at the border with exchange in mind: plasmids, biopsies from resulting mice and molecular inscriptions, whether in the form of Southern blot images or otherwise.

While the publication history of transgenic mice and their public reception are beyond the scope of this article, it is worth noting that the key groups managed to avoid much controversy by stressing their academic bona fides at a time when emerging biotech work was treated with more suspicion, and by foregrounding the skill that was required to modify mouse embryos. Individual papers and experiments raised some concerns among their scientific readers, but the multiple claims made the new mice a scientific reality; the word ‘transgenic’ was coined by Gordon and Ruddle in 1981, in a first attempt to systematize these publications.^79^

In the process of publication, as initial news appeared, competing groups were pushed to make newer claims; for instance, to show that foreign genes were working, or that they could be passed through the germline. The latter claim was made by Thomas Wagner, but more convincingly by Elizabeth Lacy and Frank Costantini, then postdocs in Chris Graham’s developmental biology lab at the University of Oxford. Both had background in molecular biology, but went to Oxford to master the various hands-on skills of embryo manipulation. Unlike the other groups here, they did not arrive at the techniques independently but learned them directly from the Yale group, going to New Haven in the 1980 Christmas break, and then reported germline transmission in transgenic mice as soon as November 1981.

Lacy and Costantini went against the early trend for divisions of labor between molecular and developmental biologists in this area. As a result, they were a force behind disseminating transgenic technology in the United States and beyond, most notably by teaching on the Cold Spring Harbor practical course on the Molecular Embryology of the Mouse, which was set up in 1983 and runs to this day. As transgenic mice were eagerly adopted in the 1980s, their production became a specialised toolset of techniques and infrastructures, and in the 1990s it became routine.

## Notes

1. For trends in the UK, largely representatives of countries with significant biomedical research, see http://www.understandinganimalresearch.org.uk/animals/numbers-animals/.

2. Rader, *Making Mice.*

3. “Products of the Year,” *Fortune*, 5 December 1988.

4. Merton, *Sociology of Science*; and Murray, “Oncomouse That Roared.”

5. Brannigan, *Social Basis of Scientific Discoveries*.

6. Bud, *Uses of Life*.

7. de Chadarevian, “Of Worms and Programmes”; Morange, “Transformation of Molecular Biology”; on developmental biology as a superdiscipline, see Hopwood, “Inclusion and Exclusion.”

8. Lévi-Strauss, *Savage Mind*, 16–17.

9. Knorr, “Tinkering toward success.”

10. Curry, *Evolution Made to Order*, 78–129. See also Lavine, “Something about X-rays.”

11. Harper, *Working Knowledge*, 74.

12. Creager and Landecker, “Technical Matters.”

13. Jacob, “Evolution and Tinkering.”

14. Ibid., 1163.

15. Morange, “French Tradition.”

16. Brenner, “Introduction,” 2. The symposium was dedicated to the retirement of Sir Gordon Wolstenholme, the Ciba Foundation’s director and chairman of the UK Genetic Manipulation Advisory Group, which explains its comparatively ambitious topic.

17. Kevles, “Renato Dulbecco”; and Scheffler, “Managing the Future.”

18. Krimsky, *Genetic Alchemy*; and Wright, *Molecular Politics*.

19. Gurdon et al., “Sexually Mature Individuals.” For Gurdon’s microinjection work, see e. g. Lane et al., “Rabbit Haemoglobin Synthesis”; and Mertz and Gurdon, “Purified DNAs.”

20. De Robertis et al., “Injected Living Cells.” See also Gurdon, “Molecular Biology in a Living Cell.” With the spread of DNA cloning, Xenopus oocytes were also envisioned as a promising system to track the elusive promoters, DNA sequences upstream of a gene that control its expression.

21. Porter and O’Connor, *Human Genetics*, 60.

22. *Developments in Cell Biology*, 6.

23. Examining the link between development and cancer was an important tradition at the Fox Chase Institute for Cancer Research; see Crowe, “Cancer, Conflict.”

24. Mintz, “Gene Expression.”

25. Mintz, “Teratocarcinoma Cells.”

26. See Kolata, *Clone*, 103–133.

27. Illmensee, “Genetic Manipulation.”

28. Dawid and Wahli, “Application of Recombinant DNA.”

29. Tim Stewart, interviewed by the author on 31 May 2012.

30. Morange, “Transformation of Molecular biology;” de Chadarevian, “Mapping Development”; and Yi, “Cancer, Viruses, and Mass Migration.”

31. Greenberg, “Bootlegging.”

32. Brinster and Arechaga, “Embryo Culture.”

33. Frank Ruddle, interviewed by the author on 8 December 2011.

34. Brinster and Arechaga, “Embryo Culture.”

35. The rabbit beta globin RNA came from Jerry Lingrel, a molecular biologist at the University of Cincinnati. Gurdon and Lingrel had published work on injecting the mouse and rabbit beta globin RNA into oocytes, that relied on the latter scientist’s novel method for purifying these molecules: Gurdon et al., “Message Stability.” The second RNA Brinster used was for a *Xenopus* 5S ribosomal component, which came from another Gurdon collaborator, Donald Brown. The *HSV-tk* gene was a gift from Carlo Croce of the Wistar Institute down the road.

36. Brinster and Arechaga, “Embryo Culture,” 868. Palmiter, interviewed by the author on 8 June 2012.

37. Brinster et al., “Translation of Globin Messenger RNA.”

38. Pellicer et al., “Introduction of a Viral Thymidine Kinase Gene.”

39. Stewart interview; Erwin Wagner, interviewed by the author on 22 February 2013.

40. Paul Berg to William Gartland, 5 February 1979, Paul Berg Papers. Available online though the National Library of Medicine Profiles in Science: http://profiles.nlm.nih.gov/ps/access/CDBBGT.pdf. Accessed on 9 August 2013.

41. Rudolf Jaenisch, interviewed by the author on 29 January 2013.

42. In his recollections, Mario Capecchi implied that he pointed Ruddle towards microinjection at a 1978 meeting in Portugal (Capecchi, “Gene Targeting”). While Capecchi’s suggestion and early results in cell culture may have been influential, Ruddle did not recall it in the interview, and as evidenced by Diacumakos’ daily planner, he may have been aware of her work as early as 1974. Elaine Diacumakos papers, RU450, Rockefeller Archives, Terrytown, NY.

43. Corvini, “Restrictions Governing DNA Research Eased.”

44. Ruddle interview.

45. Not to be confused with John Gurdon.

46. Wagner and Yun, “Fine structure.” The DNA break hypothesis has not been accepted, with current consensus stating that the super-tight packaging is achieved by replacing histones – normal chromosomal proteins that neutralise the nucleic acid negative charge and coiled DNA – with smaller protamines.

47. Thomas Wagner, interviewed by the author on 25 January 2013.

48. Keith Willison, interviewed by the author on 12 September 2013.

49. Harbers et al., “Microinjection of Cloned Retroviral Genomes,” 542.

50. Not all groups contributed to *GEIS* – for instance, Brinster did not – but the publication was then in terminal decline, with the final issue published in 1981. Crowe, “General Embryological Information Service.”

51. Brinster et al., “Mouse Oocytes,” 398.

52. U. S. Department of Health and Human Services, *Recombinant DNA Research*, 59. On the Goodman-Genentect conflict, see Kevles, “Patents, Protections, and Privileges,” 77–94.

53. Willison, “Microinjection of Mammalian Eggs,” 160.

54. Stewart interview.

55. Hopwood, “Inclusion and Exclusion.”

56. de Chadarevian, “Of Worms and Programmes,” “Mapping Development”; and Morange, “François Jacob’s Lab.”

57. Landecker, *Culturing Life*. See also Wilson, *Tissue Culture*.

58. Korzh and Strähle, “Marshall Barber.”

59. El-Badry, *Micromanipulators and Micromanipulation*, 22–72.

60. Hamburger, *Heritage of Experimental Embryology*;and Gardner, “Mouse Chimaeras.”

61. In 1979, the eminent geneticist James Crow said ‘Karl Illmensee has the embryological equivalent of a green thumb. He actually does the experiments everyone else talks about’ – cited in ‘On the Way to a Clone.’ On the other hand, Mintz was reportedly rather critical of Illmensee in private conversations once he had left Fox Chase. The golden hands argument later was pivotal to Illmensee’s defense in the face of fraud allegations about his nuclear transfer work. On micromanipulation and mammalian embryology, see also Franklin, “’Crook’ pipettes”; and *Biological Relatives*, ch. 3.

62. See note 45 above.

63. De Robertis et al., “Injected Living Cells”; andMueller et al., “Microinjection.”

64. Landecker, *Culturing Life*, 212–18.

65. See e.g. McKinnel, *Cloning*, 118: ‘Certainly, one of the virtues of the [*Xenopus* oocyte] test system is that it is natural. A nucleus is asked how it will perform in a milieu of normal and unmutilated cytoplasm.’

66. Diacumakos, “Introduction of Macromolecules,” 97–98.

67. John Gordon, interviewed by the author on 10 December 2011.

68. See note 55 above.

69. ‘In general, oocytes are very much more tolerant to injection than fertilized eggs, which are best injected at the 2-cell stage’; Willison, *Microinjection of Mammalian Eggs*.

70. Diacumakos et al., “Microsurgical Methoidology”; also emphasised in Diacumakos, “Introduction of Macromolecules.”

71. Whittingham and Biggers, “Fallopian Tube”; Whitten and Biggers, “Complete Development in Vitro”; and Biggers, “Reflections.”

72. On the origins of the recombinant DNA exchange and its crucial role in the invention of these tools, see Yi, *Recombinant University*, 83–112.

73. Pellicer et al., “Introduction of a Viral Thymidine Kinase Gene”; and Scangos et al., “Molecular Analysis.”

74. E. Wagner interview.

75. Brinster and Arechaga, “Embryo Culture”; Palmiter interview.

76. Tofano et al., “Edwin Southern.”

77. Lynch and Jordan, “Sociology of a Genetic Engineering Technique.”

78. ‘I do not look at how a knowledge claim travels from A to B, but at how the transactions made possible by the fact that A and B are distant from each other allow for the production at such a knowledge claim.’ Biagioli, *Galileo’s Instruments of Credit*, 26. I thank Robin Scheffler for pointing me in this direction.

79. Gordon and Ruddle, “Integration and Stable Germ Line Transmission.”
